# Preparation of Biodegradable and Elastic Poly(ε-caprolactone-*co*-lactide) Copolymers and Evaluation as a Localized and Sustained Drug Delivery Carrier

**DOI:** 10.3390/ijms18030671

**Published:** 2017-03-21

**Authors:** Ji Hoon Park, Bo Keun Lee, Seung Hun Park, Mal Geum Kim, Jin Woo Lee, Hye Yun Lee, Hai Bang Lee, Jae Ho Kim, Moon Suk Kim

**Affiliations:** Department of Molecular Science and Technology, Ajou University, Suwon 443-759, Korea; jhp@ajou.ac.kr (J.H.P.); acousticjazz@ajou.ac.kr (B.K.L.); hpt88@ajou.ac.kr (S.H.P.); kmg@ajou.ac.kr (M.G.K.); dlwlswlsdndn@ajou.ac.kr (J.W.L.); leeyn3679@ajou.ac.kr (H.Y.L.); hblee@ajou.ac.kr (H.B.L.); jhkim@ajou.ac.kr (J.H.K.)

**Keywords:** P(CL-*co*-LA) copolymers, drug delivery carrier, elastic polyester, biodegradation, dexamethasone

## Abstract

To develop a biodegradable polymer possessing elasticity and flexibility, we synthesized MPEG-b-(PCL-*co*-PLA) copolymers (PC*_x_*L*_y_*A), which display specific rates of flexibility and elasticity. We synthesize the PC*_x_*L*_y_*A copolymers by ring-opening polymerization of ε-caprolactone and l-lactide. PC*_x_*L*_y_*A copolymers of various compositions were synthesized with 500,000 molecular weight. The PC*_x_*L*_y_*A copolymers mechanical properties were dependent on the mole ratio of the ε-caprolactone and l-lactide components. Cyclic tensile tests were carried out to investigate the resistance to creep of PC*_x_*L*_y_*A specimens after up to 20 deformation cycles to 50% elongation. After in vivo implantation, the PC*_x_*L*_y_*A implants exhibited biocompatibility, and gradually biodegraded over an eight-week experimental period. Immunohistochemical characterization showed that the PC*_x_*L*_y_*A implants provoked in vivo inflammation, which gradually decreased over time. The copolymer was used as a drug carrier for locally implantable drugs, the hydrophobic drug dexamethasone (Dex), and the water-soluble drug dexamethasone 21-phosphate disodium salt (Dex(p)). We monitored drug-loaded PC*_x_*L*_y_*A films for in vitro and in vivo drug release over 40 days and observed real-time sustained release of near-infrared (NIR) fluorescence over an extended period from hydrophobic IR-780- and hydrophilic IR-783-loaded PC*_x_*L*_y_*A implanted in live animals. Finally, we confirmed that PC*_x_*L*_y_*A films are usable as biodegradable, elastic drug carriers.

## 1. Introduction

In the drug delivery field, biodegradable polymers can be an ideal drug carrier if they can eventually be absorbed without causing harm or adverse interactions. Additionally, an ideal drug carrier must possess the ability to remain biocompatible in the human body for controlled lengths of time for medical applications [1,2,3]. There are many types of synthetic polyesters that possess good biodegradability and biocompatibility. The United States Food and Drug Administration (FDA) has approved biodegradable poly(ε-caprolactone) (PCL), poly(l-lactic acid) (PLA), poly(glycolic acid) (PGA), and poly(lactic acid-*co*-glycolic acid) (PLGA), for tissue engineering scaffolds and drug delivery vehicles [4,5,6,7,8]. However, because of the copolymer’s poor elasticity and flexibility, it is difficult to apply to contracted and relaxed tissues such as muscle, blood vessels, and skin. To overcome this problem, it is necessary to develop a biodegradable polymer which is elastic and flexible [9,10,11,12].

Recently, the biocompatible and hydrolyzable poly(ε-caprolactone-*co*-l-lactic acid) (PC*_x_*L*_y_*A) has attracted great interest for medical applications. PCL is a semicrystalline material displaying rubbery properties, whereas PLA is a crystalline, hard, and brittle material [13,14,15,16,17]. Their PC*_x_*L*_y_*A copolymer is an attractive replacement for medical applications because of its controllable elasticity and the capacity to change the ε-caprolactone/l-lactic molar ratios and their mechanical properties. Thus, the first step in this work was the preparation and evaluation of the mechanical properties of PC*_x_*L*_y_*A copolymers with various ratios of PCL and PLA segments, to find a ratio with excellent resistance to creep [18].

In previously published papers, PC*_x_*L*_y_*A was reported to be a controllable biodegradable copolymer with excellent biocompatibility [19,20]. PC*_x_*L*_y_*A can be formulated to decompose over a period of several weeks. In addition, it has already been approved for clinical biomedical applications in the U.S. by the FDA. Thus, the second step of this work was to evaluate the biodegradability and biocompatibility of PC*_x_*L*_y_*A copolymers in vivo over a defined experimental period.

Dexamethasone (Dex) is a steroid medication, and dexamethasone 21-phosphate disodium salt (Dex(p)) is water-soluble because of its two ionic phosphate groups. Both Dex and Dex(p) are used in the treatment of many cases, a number of skin diseases, asthma, severe allergies, chronic obstructive lung disease, rheumatic disorders, and even cancer [21,22,23]. The broad range of therapeutic applications encourages the development of a Dex delivery system that is suitable for long-term administration.

We prepared a drug-loaded PC*_x_*L*_y_*A film as one of several methods of administering a sustained drug delivery system. The mechanism of biodegradable polymer film dosage is well-known for diffusion-controlled or biological degradation [24,25]. The final step of this work was to show that PC*_x_*L*_y_*A films can be loaded with Dex and Dex(p), and calibrated to achieve the release profiles at various time points.

The overall objective of the present study was to develop an elastic drug delivery carrier. First, we prepared PC*_x_*L*_y_*A copolymers with various ratios of CL and LA units and evaluated their mechanical properties to find a ratio with excellent resistance to creep. Next, we evaluated the in vivo biodegradability and biocompatibility of the PC*_x_*L*_y_*A copolymers over a defined experimental period. Finally, we showed that drug release from drug-loaded PC*_x_*L*_y_*A films could achieve a controlled release profile at various time points. This process enables a feasible development of suitable elastic drug carriers. 

## 2. Results and Discussion

### 2.1. Preparation of PC_x_L_y_A Copolymers

In the present work, the copolymerization of different ratios of caprolactone (CL) and lactic acid (LA) was performed using methoxy poly(ethylene glycol) (MPEG) as an initiator in the presence of stannous octoate at 130 °C for 24 h (Table 1). The colorless copolymers of almost quantitative yield were obtained after precipitation.

**Figure 1:**
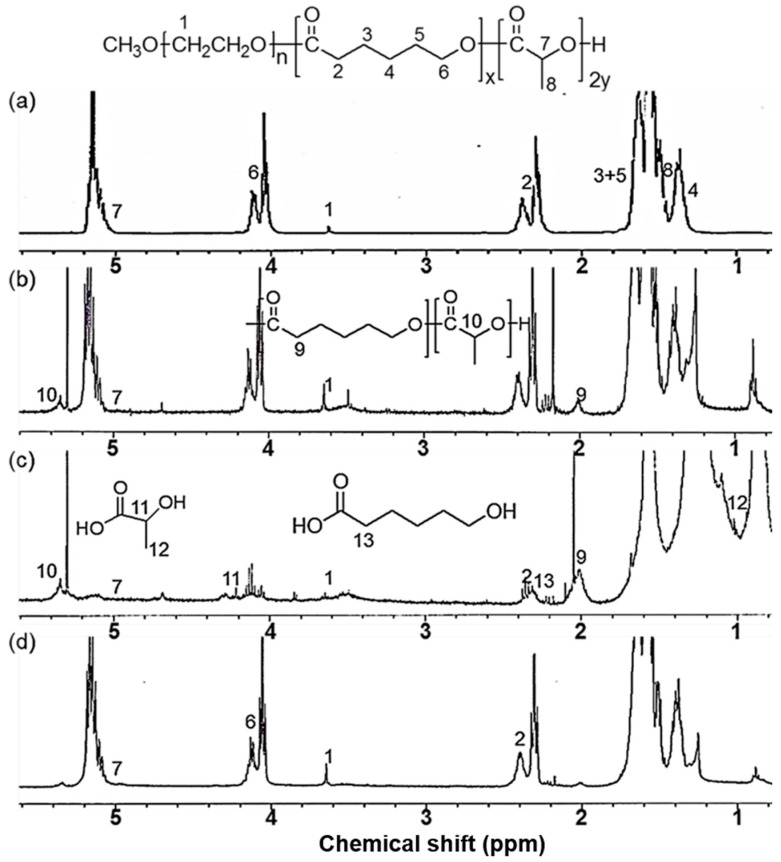
^1^H-NMR spectra of PC_5_L_5_A copolymers (**a**) before degradation and (**b**–**d**) after eight weeks in vivo implantation. (**b**) crude mixture; (**c**) *n*-hexane and ethyl ether soluble portions; (**d**) insoluble portions.

Figure 1a shows the result of the proton nuclear magnetic resonance (^1^H-NMR) spectra of PC_5_L_5_A copolymer. Since CL and LA monomers were incorporated into the PC_5_L_5_A copolymers, those two peaks were dependent on the change of feed ratio; representative ^1^H-NMR data for PC_5_L_5_A copolymer are shown (the spectra of other copolymers are not shown). Total methylene protons of MPEG used as a standard were compared with total intensities of the proton signal 1 of MPEG and the methylene protons at 2.3 and 5.2 ppm of PCL and PLA, respectively. The molecular weights of PC*_x_*L*_y_*A copolymers were calculated using the intensities of these protons. The ^1^H-NMR data also showed a good coincidence between feed and calculated ratios of the comonomers.

The prepared PC*_x_*L*_y_*A copolymers were fabricated in film form with a thickness of 200 μm for subsequent experiments.

### 2.2. Thermal Properties of PC_x_L_y_A Copolymers

**Figure 2:**
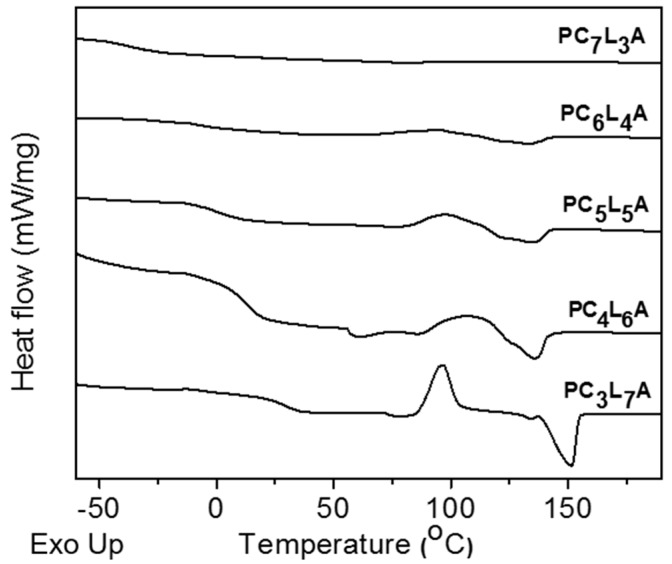
Differential scanning calorimetry (DSC) curves graph of PC*_x_*L*_y_*A copolymers.

Generally, elongation and flexibility are affected by crystalline properties [26,27]. Consequently, we investigated the PC*_x_*L*_y_*A copolymers crystalline properties using differential scanning calorimetry (DSC) (Figure 2) and X-ray diffraction (XRD) (Table 2). The degree of crystallinity of PC*_x_*L*_y_*A copolymers depends on the CL and LA content. We could not observe the melting temperatures and enthalpies of PC_7_L_3_A. PC_6_L_4_A and PC_5_L_5_A exhibited glass transition temperature (*T*_g_) and melting temperature (*T*_m_) at −3 to 2 °C and 132 to 134 °C, respectively, and low enthalpies below 6 J/g, that indicate they have both a slightly crystalline and amorphous domains. PC_4_L_6_A and PC_3_L_7_A exhibited *T*_g_ and *T*_m_ at 12 to 31 °C and 146 to 153 °C. Generally, *T*_m_ of PCL segment appeared around 60 °C and the PLA segment appeared around 150 °C, but we could only observe some *T*_m_ of the PLA. These results indicate that, due to the different reaction rates of the two monomers during the polymerizations, some LA chains are bonded to the middle part of the CL chain to show an amorphous part and the crystalline part appears due to polymerization of the remaining LA chain. These results demonstrate that the copolymers possessed semi-crystalline properties.

XRD was used to study the crystalline characteristics of the PC*_x_*L*_y_*A copolymers. The crystalline peak of PCL and PLA slightly appeared even though random copolymer, but its crystallinity was less than 10% in PC_7_L_3_A, PC_6_L_4_A, and PC_5_L_5_A.

### 2.3. Mechanical Properties of PC_x_L_y_A Copolymers

**Figure 3:**
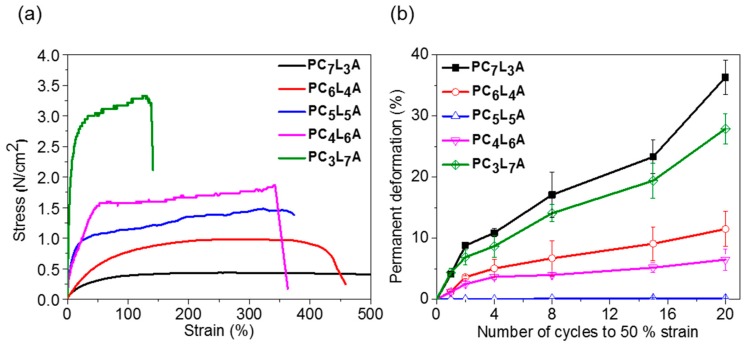
Mechanical properties of PC*_x_*L*_y_*A films. (**a**) Tensile strength; and (**b**) permanent deformation.

The elongation at break and tensile strength of PC*_x_*L*_y_*A were measured to compare their mechanical properties (Figure 3). The elongation at break and tensile strength of PC*_x_*L*_y_*A was found to depend on the monomer ratio. When the LA contents increased, the tensile strength of PC*_x_*L*_y_*A increased, but the elongation at break was decreased to increase the rigidity of the copolymer (Figure 3a). 

For medical applications involving tissues that contract and relax repeatedly, it is very important that the carrier resists creep deformation [28,29]. Cyclic tensile tests were performed to investigate the resistance to creep of PC*_x_*L*_y_*A specimens until after 20 deformation cycles to 50% elongation. Figure 3b shows the permanent deformation of PC*_x_*L*_y_*A. When the number of cycles increases, PC_7_L_3_A and PC_3_L_7_A showed an increase of permanent deformation; PC_6_L_4_A and PC_4_L_6_A showed significantly less permanent deformation, while PC_5_L_5_A recovered completely—it indicated no permanent deformation at all, even after 20 cycles.

### 2.4. In Vitro and In Vivo Degradation of PC_x_L_y_A Copolymers

**Figure 4:**
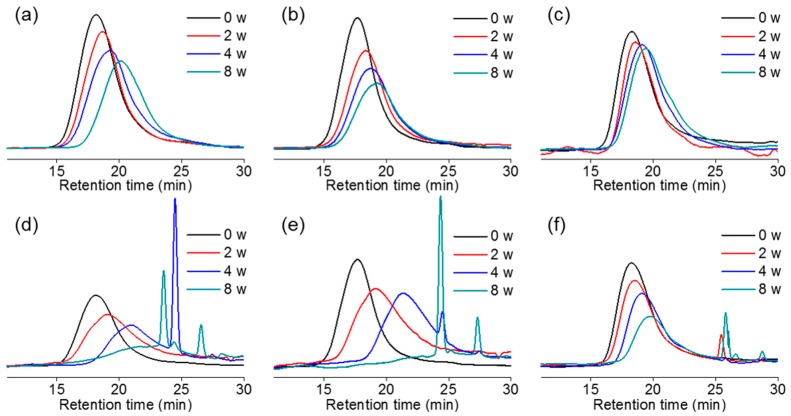
Gel permeation chromatograph (GPC) curves of PC*_x_*L*_y_*A (**a**–**c**) in vitro and (**d**–**f**) in vivo after degradation up to eight weeks (w). (**a**,**d**) PC_7_L_3_A; (**b**,**e**) PC_5_L_5_A; and (**c**,**f**) PC_3_L_7_A.

PC*_x_*L*_y_*A films in vitro degradation behavior was proved in phosphate buffer saline (PBS) at 37 °C. After eight weeks of immersion in PBS, we measured molecular weight by gel permeation chromatography (GPC) (Figure 4a–c).

**Figure 5:**
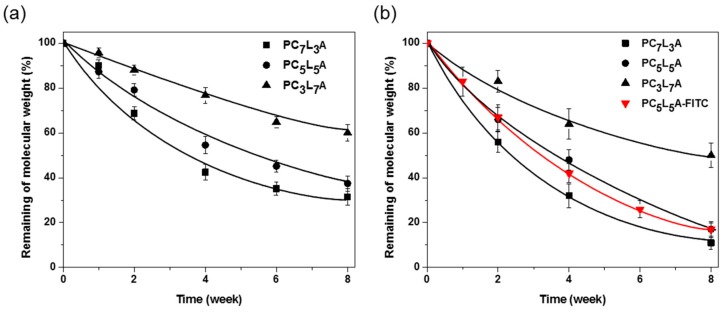
(**a**) In vitro and (**b**) in vivo PC*_x_*L*_y_*A implants degradation plot removed from rats after one to eight weeks; the red line is calculated by the fluorescence intensity in Figure 7. FITC: Fluorescein-Isothiocyanate.

Figure 5a shows the in vitro degradation of PC*_x_*L*_y_*A, determined as the time it takes to degrade the molecular weight (*M*_n_) from the original molecular weight. Molecular weights were determined by GPC compared with polystyrene and day 0 molecular weight was before the degradation as 100%. The PC*_x_*L*_y_*A degradation exhibited a large dependence on the thermal properties. As CL contents increases, the *T*_g_ of the copolymer decreases to a lower temperature and the crystallinity decreases. This means that the copolymer is becoming more amorphous. This indicates that incorporation of PCL into the copolymer segments results in faster erosion due to better accessibility of water to ester linkages.

**Figure 6:**
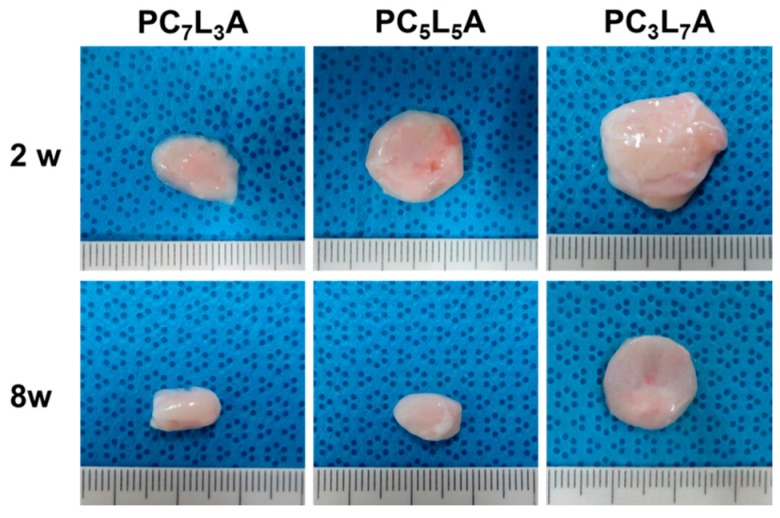
PC*_x_*L*_y_*A films removed at two and eight weeks after in vivo implantation.

For in vivo degradation, we implanted the PC_x_L_y_A films subcutaneously into Sprague-Dawley (SD) rats. PC*_x_*L*_y_*A films were allowed to develop for up to eight weeks in vivo. PC*_x_*L*_y_*A films were excised and examined at various times after implantation. Thin fibrous capsules containing a blood vessels and fibroblasts developed around the surfaces of the PC*_x_*L*_y_*A films over time (Figure 6). The PC*_x_*L*_y_*A films lasted over eight weeks, as seen in optical images, but their size was slightly decreased eight weeks post-implantation.

Figure 1a–d shows the ^1^H-NMR spectral changes of the PC_5_L_5_A films after eight weeks’ in vivo implantation. It shows a characteristic peak of the degraded polymer spectrum. Signals 11 and 12 were assignable to lactic acid, signals 1 and 13 assignable to 6-hydroxylhexanoic acid and MPEG. The four signals appeared at 4.2, 1.1, 2.1, and 3.6 ppm. These findings showed that the biodegradation products are basically lactic acid and 6-hydroxylexanoic acid which are nontoxic and biocompatible.

Figure 4d–f shows the GPC trace change in the PC*_x_*L*_y_*A films during in vivo degradation for eight weeks. A molecular weight decrease due to degradation, the retention time gradually increases through the column due to the reduced molecular weight and some of the oligomer peak shown in ^1^H-NMR at 25 min. The remaining molecular weights of PC*_x_*L*_y_*A were plotted against implantation time and are shown in Figure 5. There is parallelism of in vitro and in vivo PC*_x_*L*_y_*A film degradation. Meanwhile, the in vivo degradation rates are higher compared to in vitro biodegradation rates. Both in vitro and in vivo studies showed that biodegradation of PC*_x_*L*_y_*A co-polymers proceeded via a bulk erosion mechanism.

### 2.5. In Vivo Fluorescence Imaging

**Figure 7:**
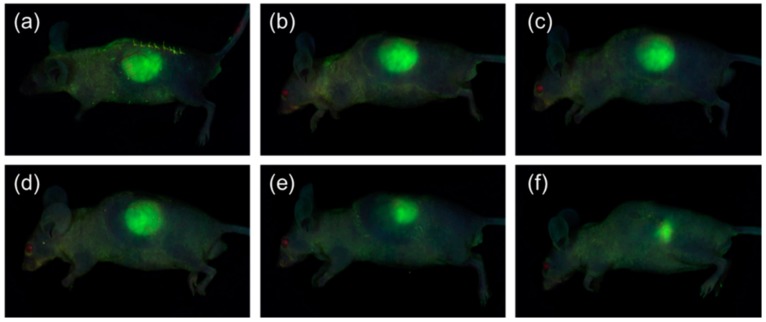
Fluorescence images of PC_5_L_5_A-Fluorescein-Isothiocyanate (FITC) (**a**) immediately after implantation; and at (**b**) one; (**c**) two; (**d**) four; (**e**) six; and (**f**) eight weeks post-implantation.

Real-time fluorescence imaging can be an effective method of evaluating the in vivo degradation. We were using PC_5_L_5_A-Fluorescein-Isothiocyanate (FITC) to monitor the real-time biodegradation using fluorescence imaging in live animals. Figure 7 shows fluorescence images acquired from nude mice following subcutaneous implantation of PC_5_L_5_A-FITC film. The fluorescence intensity of measured NEO image software version 2.3 (NeoScience, Suwon, Korea), gradually decreased over a span of eight weeks, reached a final intensity of 15% of the original intensity. Changes in fluorescence intensity for PC_5_L_5_A-FITC film obtained by Signal-to-background ratio (SBR) and displayed a similar profile and decomposition pattern (shown as a red line in Figure 5b). It is seen that the fluorescence intensity is decreased by mass loss due to the decomposition of the polymer. PC_5_L_5_A-FITC degradation occurred gradually in vivo, shown by both fluorescence imaging and GPC. This indicates that we can predict the degree of biodegradation through imaging.

### 2.6. Histological Analysis

**Figure 8:**
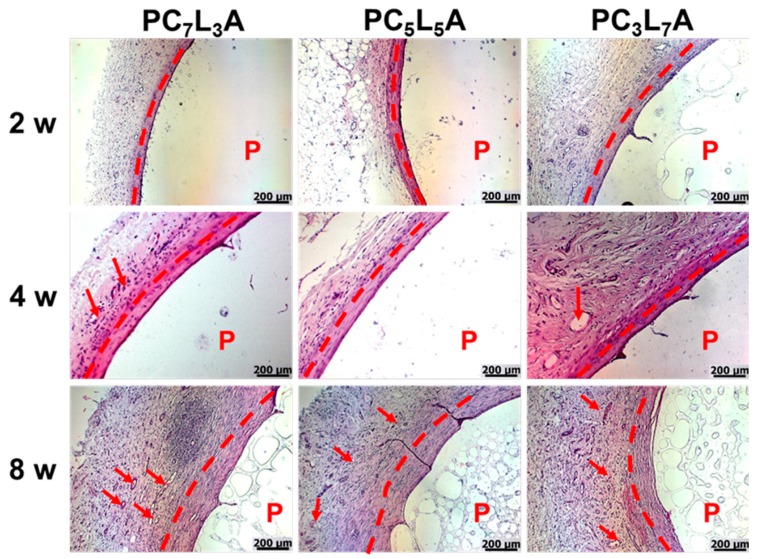
Hematoxylin and eosin (H&E) staining of PC*_x_*L*_y_*A in vivo implants removed from rats after two to eight weeks. (Arrows indicate blood vessels, scale bars represent 200 μm).

To evaluate the biocompatibility of PC*_x_*L*_y_*A, we examined histological and immunohistochemical staining by fixed tissue sections from implanted films. Hematoxylin and eosin (H&E) staining of PC*_x_*L*_y_*A films (Figure 8) revealed the distribution of polymers (empty space and pink at the bottom of the image); penetrating host cells (blue) generally has been observed as the form of rounded morphology. The PC*_x_*L*_y_*A films degraded and were filled with tissue over time, while the tissue boundary part of the polymer was stained purple or red. The degradation of the PC*_x_*L*_y_*A progresses, the host cells filled up inside the polymer, and several new blood vessels were evident in the implanted PC*_x_*L*_y_*A films.

**Figure 9:**
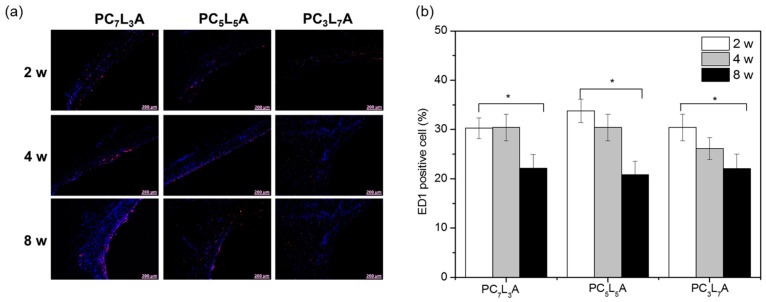
(**a**) CD68 Antibody (ED1) immunohistochemical staining of PC*_x_*L*_y_*A in vivo implants removed from rats after two to eight weeks. Scale bars represent 200 μm; (**b**) The number of ED1-positive cells on or in PC*_x_*L*_y_*A in vivo implants removed from rats after two to eight weeks.

The degree surrounding the accumulation and PC*_x_*L*_y_*A films invasion and inner inflammatory cells of the host cells were characterized by staining the tissue with CD68 Antibody (ED1) (red) to identify monocytes or macrophages, and the nuclei were stained with 6-diamino-2-phenylindole dihydrochloride (DAPI) (blue) (Figure 9a). DAPI staining indicates the number of host cells surrounding the PC*_x_*L*_y_*A films, and ED1 staining on the surface of the PC*_x_*L*_y_*A films showed the accumulation of macrophages in the surrounding tissues. 

The ED1-positive cells to determine the degree of inflammation were counted and normalized by the total stained tissue (Figure 9b). The ED1-positive cells counted on PC*_x_*L*_y_*A films comprised approximately 30% ED1-positive cells. The number of macrophages stained with ED1 decreased in PC*_x_*L*_y_*A films over time. These results demonstrate that the PC*_x_*L*_y_*A films could be used as biocompatible carriers.

### 2.7. Drug Release from Drug-Loaded PC_x_L_y_A Copolymers

The final step of this work was the development of a variety of drug delivery systems. Dex- and Dex(p)-loaded PC_5_L_5_A films were prepared by solvent casting. The quantity of drug loaded on each film, as analyzed by high-performance liquid chromatography (HPLC), was similar to the expected encapsulation of the drug. To evaluate the in vitro release, drug-loaded PC*_x_*L*_y_*A films were incubated in PBS at 37 °C for 40 days.

**Figure 10:**
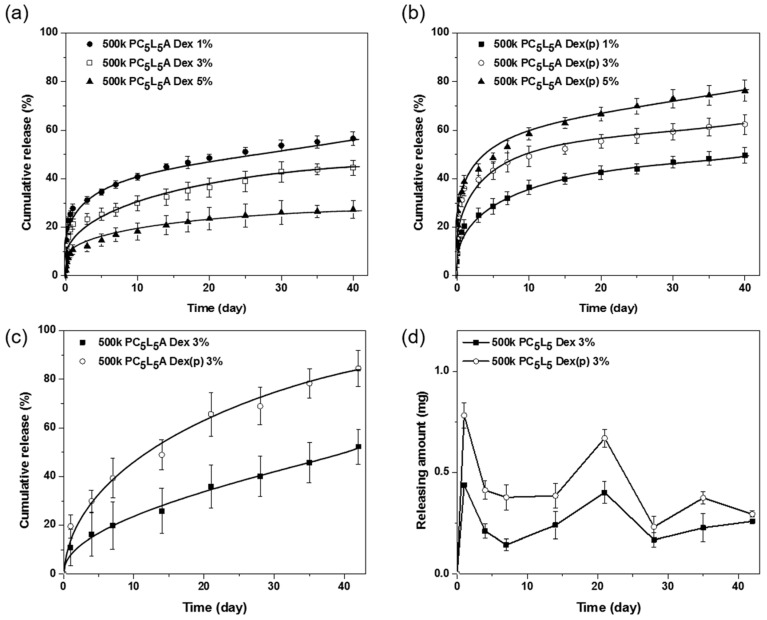
Quantities of drug released from drug-loaded PC*_x_*L*_y_*A. (**a**) Dex released in vitro; (**b**) Dex(p) released in vitro; (**c**) Dex and Dex(p) cumulative release in vivo; and (**d**) Dex and Dex(p) quantities released each day in vivo.

Figure 10a,b shows the Dex and Dex(p) release plots for an amount of cumulative released over time. The cumulative release from Dex- and Dex(p)-loaded PC*_x_*L*_y_*A films (3% concentration) in vitro at two days was approximately 20% and 35%, respectively, it was assumed that each initial burst of the drug was due to release of the drug from the surface of the PC*_x_*L*_y_*A films. After the initial burst phase, drug release was followed by a sustained-release profile for up to 40 days. The Dex release rate was increased when Dex concentration decreased from 5 to 1 *wt* % (Figure 10a). Dex molecules were likely associated via hydrophobic interactions with PC*_x_*L*_y_*A chains, interrupting water uptake into the PC*_x_*L*_y_*A films. On the other hand, the Dex(p) release rate was increased when Dex(p) concentration increased from 1 to 5 *wt* % (Figure 10b). The hydrophilic properties of Dex(p) increased water absorption by the PC*_x_*L*_y_*A films [30]. Figure 10c,d shows the Dex-loaded PC*_x_*L*_y_*A film and Dex(p)-loaded PC*_x_*L*_y_*A film plots for in vivo cumulative release up to 42 days after implantation. Both drugs showed a sustained release in vivo over 42 days; approximately 40%–80% of the Dex and Dex(p) were released from each PC*_x_*L*_y_*A films. The quantity of Dex and Dex(p) released at each time point was about 0.07–0.2 mg and 0.1–0.4 mg, respectively (Figure 10d). The in vitro and in vivo order of the drug release showed similar profiles with PC*_x_*L*_y_*A degradation. This may imply that a PC*_x_*L*_y_*A copolymer is a possible drug delivery carrier for local implantation, and suitable for delivering various drugs with adjustable degradation.

**Figure 11:**
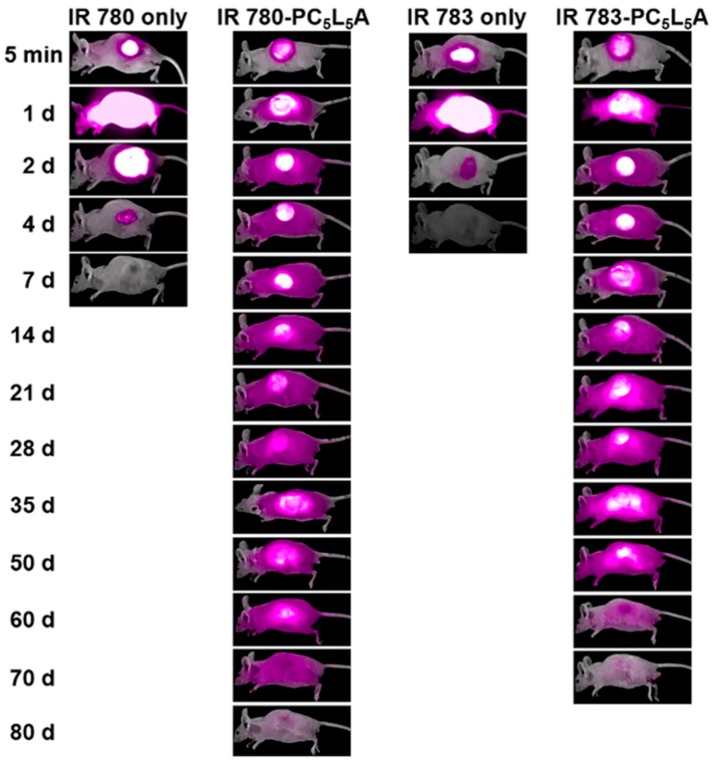
In vivo near-infrared (NIR) imaging. Exposure time is 300 ms.

Near-infrared (NIR) fluorescence imaging has been used to investigate the real-time animal in vivo sustained release of drugs from PC_5_L_5_A implants. IR-780 is hydrophobic, while IR-783 is hydrophilic. We used both to characterize the release profiles of Dex and Dex(p). NIR images were acquired after subcutaneous implantation of IR-780, IR-780-loaded PC_5_L_5_A film, IR-783, and IR-783-loaded PC_5_L_5_A film from six-week-old male nude mice (Figure 11).

In the case of IR-780 and IR-783 only, NIR fluorescence high levels were immediately observed at the injection site and diffusion was apparent after the injection. Gradually decreasing both the area of NIR fluorescence and intensity over time, and fluorescence disappeared after day 5. In contrast, high levels of NIR fluorescence were observed at the site of implantation 5 min after transplantation of the IR-780- and IR-783-loaded PC*_x_*L*_y_*A films. The fluorescence peaked on days 1 and 2. NIR fluorescence was still observed after 70 days, although both the intensity and area gradually decreased, indicating the sustained release of IR-780 and IR-783.

## 3. Materials and Methods

### 3.1. Materials

Methoxy poly(ethylene glycol) (MPEG) (Sigma-Aldrich, number-average molecular weight (*M*_n_ = 750)), and Sn(Oct)_2_ (Sigma-Aldrich, Yongin, Korea) were used as received. l-lactide (LA; Boehringer Ingelheim, Blanquefort, France) was recrystallized in ethyl acetate twice. ε-Caprolactone (CL; Sigma-Aldrich) was distilled over CaH_2_ under reduced pressure. Dex and Dex(p) were purchased from Tokyo Chemical Industry Co., Ltd. (TCI; Fukaya City, Japan). Hexafluoro-2-propanol (HFIP), IR-780, and IR-783 were purchased from Sigma-Aldrich.

### 3.2. Characterization

Spectra of proton nuclear magnetic resonance (^1^H-NMR) PC*_x_*L*_y_*A copolymers were measured using a Varian Mercury Plus 400 MHz system (Oxford Instruments, Abingdon, UK) with deuterated chloroform (CDCl_3_) in the presence of tetramethylsilane (TMS) as an internal standard. The molecular weight and dispersity of the copolymers were measured using a YL-Clarity gel permeation chromatograph (GPC) system (YL9170 RI detector) (YL instrument, Anyang-si, Gyeonggi-do, Korea) with three columns (Shodex K-802, K-803, and K-804 polystyrene gel columns) at 40 °C by polystyrene calibration and using CHCl_3_ as an eluent at a flow rate of 1.0 mL/min. The glass transition temperature (*T*_g_), melting temperature (*T*_m_), and heat of fusion (Δ*H*_m_) of the PC*_x_*L*_y_*A copolymers were determined by differential scanning calorimetry equipped (DSC; Q1000, TA Instruments, Eschborn, Germany) with Universal Analysis 2000 software from −80 to 200 °C at a heating rate of 5 °C/min for copolymers in the bulk state under a nitrogen atmosphere. The crystallinity of the various PC*_x_*L*_y_*A copolymers was measured by conducting X-ray diffraction (XRD; High Resolution X-ray Diffractometer, Ultima III, Rigaku, Japan). The crystallinity degree was calculated as the ratio of crystalline peak areas to the total area under the scattering curve.

### 3.3. Synthesis of PC_x_L_y_A Copolymers

The typical polymerization process produces PC_5_L_5_A with a CL/LA ratio of 50/50 and molecular weight of 500 kg/mol. MPEG (0.012 g, 0.016 mmol) with number-average molecular weight (*M*_n_) of 750 g/mol, and toluene (75 mL) were added into a flask. Water was removed from MPEG and toluene by azeotropic distillation. Under a dry nitrogen stream, toluene was then distilled to give a final volume of 40 mL. CL (3.5 g, 31 mmol) and LA (4.5 g, 31 mmol) were added to the MPEG solution at room temperature, followed by the addition of 0.2 mL of Sn(Oct)_2_ solution (0.1 M in dried toluene). After stirring for 24 h at 130 °C, the reaction mixture was poured into a mixture of *n*-hexane and ethyl ether (*v*/*v* = 4/1) to precipitate a polymer, which was separated from the supernatant by decantation, dissolved in CH_2_Cl_2_, and then filtered. The resulting polymer solution was concentrated on a rotary evaporation and then dried in a vacuum to yield a colorless polymer. In the same way, other PC*_x_*L*_y_*A copolymers were prepared. The ratios of the PCL and PLA segments and molecular weights of the copolymers were determined using ^1^H-NMR spectra by comparing the intensity of total MPEG methyl proton signals at δ = 3.38 ppm, the methylene proton signals of PCL at δ = 2.3 ppm, and methine proton signals of PLA at δ = 5.2 ppm.

### 3.4. Synthesis of PC_x_L_y_A Copolymer with Fluorescein-Isothiocyanate (FITC) (PC_5_L_5_A-FITC)

PC_5_L_5_A (4 g, 0.008 mmol) and toluene (80 mL) were introduced into a flask. Water was removed from the PC_5_L_5_A and toluene by azeotropic distillation. Under a dry nitrogen stream, toluene was then distilled to give a final volume of 40 mL. FITC (4.1 mg, 0.096 mmol) was added to the PC_5_L_5_A solution at room temperature, followed by the addition of 0.1 mL of Sn(Oct)_2_ solution (0.1 M in dried toluene). After stirring at 130 °C for 24 h, reaction mixture was poured into a mixture of ethyl ether, *n*-hexane and methanol (*v*/*v*/*v* = 2/1/1) to precipitate a polymer. The precipitated polymers were dissolved in CH_2_Cl_2_, and filtered. The resulting polymer solution was concentrated by rotary evaporation and dried in a vacuum to yield a yellow polymer.

### 3.5. Preparation of PC_x_L_y_A Films

Each PC*_x_*L*_y_*A copolymer was dissolved at a concentration of 20 *wt* % in dichloromethane. The PC*_x_*L*_y_*A solution was spread on a polyethylene film. The solvent was slowly evaporated at 5 °C for four days, and the PC*_x_*L*_y_*A films were then dried at room temperature in a vacuum oven for two days. PC*_x_*L*_y_*A films were cut into discs with a diameter of 16 mm and thickness of 200 μm. These discs weighed approximately 25 mg (25 ± 5 mg).

### 3.6. Mechanical Testing of PC_x_L_y_A Films

PC*_x_*L*_y_*A films were prepared at dog-bone shape (the specimens were made with 0.5 mm thickness, the total area of the film was 15 × 50 mm^2^, and the middle section measured 5 × 10 mm^2^). The mechanical properties of the polymers were measured by Universal Testing Machine (H5KT, Tinius Olsen, Horsham, PA, USA) at a cross-head speed in vertical direction at a rate of 3 mm/min strain velocity at room temperature by using 50 N load cells up to specimen break.

Under the same operating conditions, it was subjected to a tensile test of the cyclic loading. The test specimens 20 cycles, was deformed up to 50% elongation. As a result of the creep in the cyclic test, permanent set (the permanent deformation) was the measured value.

### 3.7. In Vitro Degradation Test

Each disc was soaked in 10 mL of PBS in a conical tube (50 mL), it was incubated at 37 °C with shaking at 100 rpm for eight weeks. At predetermined time intervals, conical tubes were retrieved and freeze-dried for eight days. After lyophilization, changes of *M*_n_ were measured by a GPC system.

### 3.8. In Vivo Implantation

A total of 36 eight-week-old Sprague-Dawley (SD) rats (270–350 g) were used according to the guidelines that have been approved. Each rat was anesthetized with Rompun and Zoletil (1:1 ratio, 1.5 mL/kg). The rats were randomly assigned to three groups SD rats (two, four, and eight weeks) and used for the GPC measurements and histological analyses. All animals were treated in accordance with the Institutional Animal Experiment Care and Use Committee of at Ajou University School of Medicine (Approval No. 2014-0051, date 03-03-15). The copolymer discs, sterilized using ethylene oxide gas, were subcutaneously implanted on the back side and allowed to develop; they were biopsied at different time points in vivo over eight weeks. In each sampling point after transplantation, the rats were sacrificed and the film implants removed. Each film implant was placed into a test tube and added CH_2_Cl_2_ (1 mL) to dissolve a portion of the copolymer implant. Then, 1 mL of deionized water (DW) was added to solubilize the subcutaneous tissue. The resulting mixture was sonicated for 90 min at 25 °C. The CH_2_Cl_2_ solution was collected and removed, and the remaining copolymer was freeze-dried until it reached a constant weight and was measured by GPC. The molecular weights (*M*_n_) of the in vivo-degraded PC*_x_*L*_y_*A were measured at the maximum GPC peaks. The degree of decomposition of PC*_x_*L*_y_*A was calculated using the relative ratio of the molecular weights determined during the eight-week experiment and on the initial day.

### 3.9. Histological Analysis

At two, four, and eight weeks after implantation, film implants were removed and were immediately fixed with 10% formalin for four days. The fixed tissues were dehydrated with 100% ethanol and embedded in paraffin. The embedded specimens were sectioned along the longitudinal axis of the implant (4 μm), and the representative sections were stained with hematoxylin and eosin (H&E) and examined by light microscopy.

For 6-diamino-2-phenylindole dihydrochloride (DAPI) and ED1 staining, the slides were deparaffinized and hydrated. Next, the slides were rinsed and washed with PBS-T (0.05% Tween 20 in PBS) and blocked with 5% horse serum (HS; Gibco, Invitrogen, Auckland, New Zealand) and 5% bovine serum albumin (BSA; Bovogen, Keilor East, Victoria, Australia) in PBS at 37 °C for 1 h. The sections were subsequently exposed to a mouse anti-rat CD68 antibody (ED1; Serotec, Oxford, UK) and incubated overnight at 4 °C. After rinsing with PBS-T, the slides were incubated with the secondary antibody (goat anti-mouse Alexa Fluor 594; Invitrogen, San Diego, CA, USA) in the dark at room temperature. After 3 h, the slides were washed again PBS-T and finally counter-stained with DAPI (Sigma-Aldrich, St. Louis, MO, USA), and developed with fluorescent mounting solution (Dako, Carpinteria, CA, USA). Immunofluorescent images were obtained with an Axio Imager A1 (Carl Zeiss Microimaging GmbH, Göttingen, Germany) equipped with Axiovision Rel. 4.8 software (Carl Zeiss Microimaging GmbH). Imaging and quantification of each slide were measured at three random different positions. The separator between the film and the host tissue were determined using differential interference contrast (DIC) optical microscope image of the previous acquisition of immunofluorescence image.

### 3.10. Preparation of Drug-Loaded Polymer Films

Drug-loaded PC*_x_*L*_y_*A films were prepared using solvent casting. 1, 3, 5 *wt* % drug was added to the copolymer solution (20 *wt* %). Dex with copolymer was dissolved in methylene chloride (MC) and Dex(p) with copolymer was dissolved in HFIP. The mixtures were cast on a polyethylene film and allowed to slowly dry for four days at 10 °C, and then they were dried in a vacuum oven at room temperature for two days. Copolymer films were cut into discs with 16 mm diameters and 200 μm thicknesses weighing 28 mg (28 ± 4 mg).

### 3.11. In Vitro Release

Drug-loaded PC*_x_*L*_y_*A films were immersed in 10 mL PBS in 20 mL vials and shaken at 100 rpm and 37 °C for up to 40 days. For each experiment, 1 mL of the sample was extracted from the vials at determined time intervals; 1 mL fresh PBS was immediately added into the vials. The amount of Dex and Dex(p) was analyzed using an HPLC system (Agilent 1200 series, Waldbronn, Germany) equipped with detection at 220 nm. The RP18 column (3.9 mm × 150 mm, 5 μm) for Dex and Hypersil C18 column (4.6 mm × 250 mm, 5 μm) for Dex(p) were used. The mobile phase consisted of a deionized water (DW) and acetonitrile (50:50, *v/v*) mixture for Dex and 60:40, *v*/*v* mixture for Dex(p). The mobile phase was eluted at a flow rate of 1.0 mL/min. Three independent release experiments were performed for each drug-loaded PC*_x_*L*_y_*A film composition. Eluents were identified by comparing their peaks to the retention time of pure standards and quantified by ultraviolet (UV) absorption peak area of 254 nm.

### 3.12. In Vivo Release

Drug-loaded PC*_x_*L*_y_*A films were sterilized using ethylene oxide gas. Eight-week-old SD rats (270–350 g), divided into eight groups (1, 4, 7, 14, 21, 28, 35, and 42 days) were anesthetized using Zoletil (Virbac, Pukete, New Zealand) and Rompun (Bayer, Leverkusen, Germany) (1:1 ratio, 1.5 mL/kg). The films (*n* = 4 for each SD rat) were implanted subcutaneously under the dorsal skin, releasing drug in vivo over 42 days. At each of the sampling points after post-implantation the rats were sacrificed, and the film implants were dissected individually and removed. The amount of drug that is released from each of the film was analyzed using HPLC.

### 3.13. In Vivo Fluorescence Imaging

At selected times, side-view images of the PC_5_L_5_A-FITC films implanted in the mice were taken using an imaging instrument (FOBI, NeoScience, Suwon, Korea) equipped with NEO image software (NeoScience, Suwon, Korea).

### 3.14. In Vivo Near-Infrared (NIR) Imaging

IR-780- and IR-783-loaded PC*_x_*L*_y_*A films were prepared using solvent casting. 1, 3, 5 *wt* % NIR was added to the copolymer solution (20 *wt* %). IR-780 was dissolved in methylene chloride (MC) and IR-783 was dissolved in HFIP. NIR-loaded PC*_x_*L*_y_*A were implanted subcutaneously in the left dorsum of six-week-old male nude mice (anesthetized with Zoletil and Rompun). At selected times, side-view images of the mice were collected using a FOBI imaging instrument equipped with NEO image software.

### 3.15. Statistical Analysis

DSC data, ED1, assays, and tensile strength values of PC*_x_*L*_y_*A films were recorded in independent experiments (*n* = 3 for each data point); all values are reported as mean ± standard deviation (SD). The results were analyzed with one-way ANOVA using the Prism 3.0 software package (GraphPad Software Inc., San Diego, CA, USA); *p*-values lower than 0.01 or 0.05 were considered statistically significant.

## 4. Conclusions

In the present study, we prepared PC*_x_*L*_y_*A copolymers to investigate their feasibility as elastic drug carriers for drug delivery systems. PC*_x_*L*_y_*A copolymers showed good mechanical properties, and especially low creep rates. In addition, PC*_x_*L*_y_*A copolymers exhibited in vivo biodegradability and biocompatibility for a defined experimental period and induced only a modest inflammatory response. Drug-loaded PC*_x_*L*_y_*A copolymers exhibited sustained drug release both in vitro and in vivo. These results indicate that PC*_x_*L*_y_*A copolymers can serve as a suitable drug delivery carrier with elastic properties in the local implant.

## Figures and Tables

**Table 1:** Synthesis of MPEG-b-(PCL-co-PLA) (PC*_x_*L*_y_*A) copolymers.

Polymer	Molar Ratio (%) (CL/LA) ^a^	Yield (%) ^b^	*M*_n_^a^	*M*_W_/*M*_n_^c^
PC_7_L_3_A	70/30	90	530,000	1.7
PC_6_L_4_A	61/39	89	516,000	1.6
PC_5_L_5_A	51/49	92	498,000	1.4
PC_4_L_6_A	38/62	93	521,000	1.7
PC_3_L_7_A	28/73	91	512,000	1.5

Initiator is methoxy poly(ethylene glycol) (MPEG); Condition: [Sn(Oct)_2_]/[Initiator] = 1.2; ([ε-CL] + [LA])/[toluene] = 2.8 M, 130 °C, 24 h; ^a^ Determined by proton nuclear magnetic resonance (^1^H-NMR) spectroscopy; ^b^ *n*-Hexane/diethyl ether (4/1) insoluble part; ^c^ Measured by gel permeation chromatography (GPC) (Based on polystyrene standards). CL: caprolactone; LA: lactic acid; *M*_n_: the number average molecular weight; *M*_W_: the weight average molecular weight.

**Table 2:** Thermal properties of poly(ε-caprolactone-co-lactide) (PCLA) copolymers.

Polymer	*T*_g_ (°C) ^a^	*T*_m_ (°C)	Δ*H* (J·g^−1^)	*X*_c_^b^
PC_7_L_3_A	−31.7	-	-	7.9
PC_6_L_4_A	−2.8	132.3	1.3	8.7
PC_5_L_5_A	1.7	134	5.9	9.6
PC_4_L_6_A	12.25	136	13.2	12.7
PC_3_L_7_A	31.7	153.6	26.8	19.7

^a^ Measured by differential scanning calorimeter (DSC); ^b^ *X*_c_ was calculated as the ratio of crystalline peak areas to the total areas under the scattering curve. *T*_m_: melting temperature; *T*_g_: glass transition temperature; Δ*H*: heat of fusion; *X*_c_: crystallinity.
